# Clinical and laboratory parameters as predictors of mortality in patients with chronic liver disease presenting to emergency department- a cross sectional study

**DOI:** 10.1186/s12245-024-00647-9

**Published:** 2024-06-13

**Authors:** Salva Ameena M S, Vempalli Nagasubramanyam, Anand Sharma, Nidhi Kaeley, Bharat Bhushan Bhardwaj, Poonam Arora, Althaf Assis, Takshak Shankar, Hari Prasad, Mukund Rajta, Ashwani Pundir

**Affiliations:** 1 Department of Emergency Medicine, All India Institute of Medical Sciences Rishikesh, Rishikesh, Uttarakhand 249203 India; 2 Department of Emergency Medicine, All India Institute of Medical Sciences, Gorakhpur, Uttar Pradesh 273008 India; 3 Department of Gastroenterology, All India Institute of Medical Sciences Rishikesh, Rishikesh, Uttarakhand 249203 India; 4 Department of Community Medicine, J. N. Medical College, Aligarh Muslim University, Aligarh, 202001 India

**Keywords:** Chronic liver disease, Child-pugh score, Cirrhosis, Hepatic encephalopathy

## Abstract

**Background:**

The World Health Organization (WHO) reports that Asia and Africa have the highest Chronic Liver Disease (CLD) mortality rate. Cirrhosis, responsible for 22.2 fatalities per 100,000 people, is India’s 10th most common cause of mortality. The increasing prevalence of chronic liver disease necessitates a study to identify predictive factors for patients who visit the emergency department. Identifying elements that enhance the predictive value of mortality in unstable patients with CLD complications is important in emergency departments. This study aims to determine Clinical and Laboratory Parameters as mortality predictors in adult chronic liver disease patients.

**Methodology:**

The study was conducted at the emergency department of a tertiary healthcare center in Northern India. Patients with chronic liver disease above 18 years of age who satisfied the inclusion criteria were clinically evaluated. Clinical and demographic details were collected, and data was analyzed.

**Results:**

Two hundred thirty-six patients were enrolled. The mean age was 50.77 ± 14.26 years. 78.4% of the participants were men. Abdominal distension, affecting 59.7% of patients, was the most common presenting ailment, followed by melena and hematemesis, affecting 41.9% and 32.6%, respectively. The mean stay in the emergency department was 10.29 ± 8.10 h. Refractory septic shock, the leading cause of mortality, accounts for 69.2% of all deaths, alongside grade 4 hepatic encephalopathy and massive Upper Gastrointestinal (UGI) bleeding, as identified in our study. Factors such as altered mental sensorium, high respiratory rate, low SpO2, increased heart rate, low systolic blood pressure, low diastolic blood pressure, and low Glasgow Coma Scale (GCS) on Emergency Department (ED) arrival are significantly associated with mortality.

**Conclusions:**

Chronic liver disease, a prevalent condition in India, most commonly seen in middle aged men and lower socioeconomic groups. The parameters independently associated with mortality in our study were presence of altered mental sensorium, Glasgow coma scale, Child Pugh class and need for ICU admission. Understanding the presentation pattern, and mortality predictors can help ED physicians in managing acute events and follow-ups.

## Background

Chronic liver Disease (CLD) refers to liver function decline lasting over six months. This includes clotting and protein synthesis, detoxification, and bile excretion. CLD causes inflammation, parenchymal damage, and regeneration, resulting in cirrhosis and fibrosis [1]. Chronic liver disease has many causes, including toxins, alcohol, infection, autoimmune diseases, genetics, and metabolic issues. The final stage, cirrhosis, results in liver architecture disruption, nodule development, circulatory system reorganization, neo-angiogenesis, and extracellular matrix deposition and changes develop over weeks to years [1].

Cirrhosis presents with various clinical symptoms depending on the underlying cause. Chronic liver disease is highly prevalent and a leading cause of death globally, with an estimated 1.5 billion cases worldwide and a frequency of 20.7 per 100,000 people, up 13% since 2000 [2]. Mortality from CLD is increasing in low-income and low-middle-income countries such as Asia and Africa, with India being one of the worst affected countries, where cirrhosis is responsible for 22.2 fatalities per 100,000 people [3].

Cirrhosis has two phases: preclinical and clinical. The preclinical stage lasts for several years, but once clinical symptoms like ascites, encephalopathy, and variceal hemorrhage occur, the disease’s remaining course is shorter and often fatal [4]. The disease is considered decompensated when a patient experience one of the main consequences of cirrhosis, such as ascites, jaundice, encephalopathy, or gastrointestinal hemorrhage. Without a liver transplant, the yearly mortality rate after initial decompensation is around 10% [5].

As chronic liver disease (CLD) progresses, patients experience complications that worsen liver-related morbidity and mortality. Decompensating events occur in 4–12% of patients with cirrhosis annually, the most common being ascites, variceal hemorrhage, and hepatic encephalopathy [2]. Researchers found that 43% of patients with CLD were admitted to the intensive care unit (ICU) directly from the emergency department, indicating a high incidence of critical patients among ED visitors [6]. Identifying elements that improve the prognostic value of mortality in unstable patients with CLD complications is crucial for emergency departments. This study aimed to discover laboratory and clinical parameters that predict mortality in patients with CLD who visited the ED.

With the rate of chronic liver disease increasing, a study of the predictive factors for those patients who visited the ED is warranted. Geographical, socioeconomic, and etiological factors, as well as age and ethnicity, can influence the profile of chronic liver disease and related mortality. There are only a few studies on the clinical and laboratory parameters as predictors of mortality in chronic liver disease in the Indian population, specifically regarding emergency presentations. This study aims to determine Clinical and Laboratory Parameters as mortality predictors in adult chronic liver disease patients.

## Methods

### Study design and settings

This cross-sectional study was conducted in the Emergency Department of All India Institute of Medical Sciences, Rishikesh, Uttarakhand, between November 2021 to April 2023 (patient recruitment period from January 2021 to January 2022). Institute ethics committee approval was obtained before the commencement of the study (IEC approval number: AIIMS/IEC/21/600).

### Selection of patients

#### Inclusion criteria

1. Patients 18 years of age or above.
2. Patients previously diagnosed with chronic liver disease or those diagnosed with chronic liver disease during the hospital stay by treating clinician and team as per standard clinical, biochemical, radiological, endoscopic, or histological criteria.

#### Exclusion criteria

1. Patients/attendees not giving consent for the study.
2. Patients diagnosed with hepatocellular carcinoma.

### Sample size

Sample size is calculated by the formula, n = [DEFF*Np(1-p)]/ [(d2 /Z21-a/2*(N-1) + p*(1-p)]. The expected number of chronic liver diseases during the study period is 600. Hypothesized percentage of frequency of outcome factor in population taken as 50%+/-5%. The sample size was calculated as 235 with an absolute precision of 5% with a confidence level of 95%. In this study, 236 patients were recruited.

### Operational definitions

“Chronic liver disease: Chronic liver disease (CLD) will be classified into cirrhosis and non-cirrhotic CLD” [7].

“Cirrhosis of the liver with portal hypertension will be diagnosed based on standard clinical features (presence of ascites), radiological evidences (shrunken liver, dilated portal vein with Periportal or other collaterals), endoscopic evidence (presence of esophageal/gastric/ectopic varices and/or portal hypertensive gastropathy) and biopsy features of cirrhosis (in patients in whom biopsy is indicated)” [7].

“Non-cirrhotic CLD: This was defined as an inclusive category for all patients that presented with a history and/or evidence of chronic liver dysfunction in the form of ultrasound-proven hepatomegaly and/or persistent abnormality (more than six month duration) in liver function test (elevated liver enzymes and/or jaundice) but did not qualify to be labeled as cirrhosis or hepatocellular carcinoma” [7].

Ascites: Ascites was either overt or detected during ultrasound screening.

Spontaneous bacterial peritonitis: Ascitic fluid polymorphonuclear cell count of more than 250 cells/ cubic mm and/or ascitic fluid culture positivity [8].

Hepatic encephalopathy: This will be diagnosed as per West Haven criteria [8].

The Modified Kuppuswamy socioeconomic scale is used to document the patient’s socioeconomic status [9].

### Clinical evaluation

Patients with chronic liver disease over 18 years old and meeting inclusion criteria are selected for the study. Informed consent was obtained from patients or relatives. Demographic information, including name, age, sex, and medical history were collected. The patient evaluation form was used to collect information about presenting complaints, examination findings, investigations, and treatment received. Clinical presentation patterns and associated complications were recorded. Patient information, including ICU/non-ICU admission and critical events until discharge or death, is gathered from hospital records.

### Statistical analysis

Data were entered in Excel spreadsheet. SPSS v23 (IBM Corp.) was used for data analysis. Descriptive statistics was elaborated in the form of means/standard deviations and medians/ interquartile range (IQR) for continuous variables, and frequencies and percentages for categorical variables. Association between variables where one is continuous, and one is categorical was explored using independent sample ‘t-test when the categorical variable has two categories, and one-way ANOVA (Analysis of variance) when it has more than two categories. Linear correlation between two continuous variables will be explored using Pearson’s correlation (if the data was normally distributed) and Spearman’s correlation (for non-normally distributed data). Statistical significance was kept at p < 0.05.

## Results

### Demographics of study participants

After obtaining informed consent, 236 eligible patients were included in the study with a mean age of 50.77 ± 14.26 years. Most participants were male (78.4%) and illiterate (26.7%). Diabetes Mellitus was the most common comorbidity noted (21.2%) apart from CLD. 108 (45.8%) had a history of alcohol consumption, and 31 (13.1%) were smokers. The study found that abdominal distension was the most common presenting complaint, affecting 59.7% of participants. Hematemesis and melena were also reported, affecting 41.9% and 32.6% of participants. The mean hemoglobin value was 9.20 ± 2.38 (g/dL), while mean platelets were 117.85 ± 59.74 × 103/micro-L.

### Emergency department (ED) intervention and outcomes of participants

53.8% underwent ascites paracentesis, and 49.6% received blood component transfusions. 11.4% required intubation. Ninety patients had upper gastrointestinal (UGI) endoscopy or endoscopic interventions (Table 1).

**Table 1:** Emergency department intervention of participants

Intervention	Number (%)
Ascites Paracentesis	127 (53.8%)
IV Fluid Resuscitation	148 (62.7%)
Blood Component Transfusion	117 (49.6%)
UGI Endoscopy/ Endoscopic Interventions	90 (38.1%)
Oxygen Requirement	30 (12.7%)
Intubation	27 (11.4%)
**Emergency department disposition**	**Number (%)**
Discharged from	68 (28.8%)
Admitted To Ward	43 (18.2%)
Left against medical advice (LAMA)	41 (17.4%)
Admitted To Intensive care unit (ICU)	35 (14.8%)
Death	31 (13.1%)
Referral	25 (10.6%)
Abscond From emergency department	6 (2.5%)

### Outcome and mortality

The mean stay in the emergency department was 10.29 ± 8.10 h. Thirty-five individuals (14.8%) were given an Intensive care unit (ICU admission), and 43 participants (18.2%) were admitted to the ward. Of the participants, 68 (20.8%) were discharged from the emergency department, and 31 (13.1%) participants succumbed to death in the emergency room (Table 2). The mean hospital stay was 6.33 ± 4.10 days, and the mean length of stay in the ICU was 4.21 ± 4.03 days. 9 (3.8%) received dialysis, and 40 or 16.9% of the participants died while hospitalized (Table 2). Refractory septic shock, the leading cause of mortality, accounts for 69.2% of all deaths, alongside grade 4 hepatic encephalopathy and massive UGI bleeding, as identified. (Table 2). Notably, during ED stay, altered mental sensorium, high respiratory rate, low SpO2 (%), increased heart rate, low systolic blood pressure, low diastolic blood pressure, and low GCS all had a statistically significant association with mortality, as indicated by Table 3.

**Table 2:** Outcomes and causes of mortality in participants

Hospital outcome	Mean ± SD OR number (%)
Length of ICU stay (Days)	4.21 ± 4.03
Length of hospital stay (Days)	6.33 ± 4.10
Mortality during hospital stay (Yes)	40 (16.9%)
Need for ventilator support (Yes)	34 (14.4%)
Need for Oxygen support (Yes)	15 (6.4%)
Need for dialysis (Yes)	9 (3.8%)
**Cause of death**	**Number (%)**
Refractory Septic Shock	27 (69.2%)
Hepatic encephalopathy Grade 4	14 (35.9%)
Massive UGI Bleed/Hypovolemic Shock	9 (23.1%)
Acute respiratory distress syndrome	2 (5.1%)

**Table 3:** Association of clinical and biochemical parameters with mortality

Parameters	Mean ± SD or Number (%)		*P* value
	Not survived (*n* = 31)	Survived (*n* = 205)	
Altered mental sensorium	14 (45.2%)	44 (21.5%)	0.004
Respiratory Rate (CPM)	24.55 ± 6.70	20.60 ± 3.87	< 0.001
SpO2 (%)	88.39 ± 13.35	95.62 ± 4.39	< 0.001
Heart Rate (BPM)	104.81 ± 19.89	94.30 ± 17.93	0.001
Systolic BP (mmHg)	83.65 ± 18.98	109.81 ± 20.22	< 0.001
Diastolic BP (mmHg)	49.52 ± 18.20	69.20 ± 14.05	< 0.001
GCS	10.06 ± 4.40	14.14 ± 2.06	< 0.001
RBS (mg/dL)	117.48 ± 47.35	136.15 ± 51.43	0.037
Hemoglobin (g/dL)	7.82 ± 2.28	9.41 ± 2.32	0.001
HCT (%)	24.54 ± 6.24	28.01 ± 6.49	0.003
Platelets (x 10^3^/micro L)	99.02 ± 57.54	120.70 ± 59.68	0.006
TLC (X 10^3^/micro L)	14.65 ± 7.50	10.96 ± 10.59	< 0.001
pH	7.29 ± 0.12	7.37 ± 0.09	< 0.001
Bicarbonate (mmol/L)	14.41 ± 5.48	18.42 ± 4.50	< 0.001
Lactate (mmol/L)	5.88 ± 3.94	2.80 ± 2.34	< 0.001
PT (seconds)	24.74 ± 11.15	18.62 ± 13.47	< 0.001
INR	2.20 ± 0.90	1.57 ± 0.63	< 0.001
Blood Urea (mg/dL)	85.84 ± 46.07	61.49 ± 40.06	0.001
S. Creatinine (mg/dL)	2.19 ± 1.30	1.73 ± 4.02	< 0.001
AST (U/L)	200.54 ± 493.99	96.78 ± 229.90	0.001
ALT (U/L)	92.23 ± 116.87	51.47 ± 43.30	0.008
S. Albumin (g/dL)	2.24 ± 0.62	2.55 ± 0.47	0.002
Intubation	14 (45.2%)	13 (6.3%)	< 0.001
Oxygen Requirement	10 (32.3%)	20 (9.8%)	0.002
IV Fluid Resuscitation	27 (87.1%)	121 (59.0%)	0.003

*ALT* Alanine aminotransferase, *ALP* Alkaline phosphatase, *ANC>* Absolute neutrophil count, *AST* Aspartate aminotransferase, *BPM* Beats per minute, *BP* blood pressure, *CPM* Counts per minute, *GCS* Glasgow coma scale, *INR* International Normalised Ratio, *RBS* Random blood sugar, *TLC* Total leukocyte count

The Table 4 shows the logistic regression model to identify predictors of mortality in cases with chronic liver disease/cirrhosis. Patient parameters found to be statistically significant on univariate analysis were entered into the model. It was found altered mental sensorium, Glasgow coma scale, Child Pugh class and need for ICU admission were found to be statistically associated with mortality. The regression model had a pseudo-R square value of 0.777, implying 77.7% of variance associated with mortality of patient was explained by the parameters entered into the model.

**Table 4:** Multivariate logistic regression to identify parameters as predictor of mortality

Patient parameters found to be significantly associated in univariate analysis	Logistic regression coefficient (95% CI)	*p*-value (Dependent variable outcome in favour of mortality)
Age of the patient	0.974 (0.906–1.048)	0.492
Sex of the patient	0.081 (0.002–2.575)	0.155
Altered mental sensorium	3.013 (1.379–6.587)	0.023
Respiratory Rate (CPM)	1.13 (0.882–1.446)	0.332
SpO2 (%)	0 (0–19087.345)	0.299
Heart Rate (BPM)	0.966 (0.9–1.036)	0.338
Systolic BP (mmHg)	1.001 (0.919–1.092)	0.966
Diastolic BP (mmHg)	0.934 (0.829–1.052)	0.263
GCS	0.267 (0.123–0.581)	0.001
RBS (mg/dL)	0.981 (0.959–1.003)	0.090
Haemoglobin (g/dL)	0.425 (0.172–1.048)	0.063
HCT (%)	1.164 (0.881–1.537)	0.285
Platelets (x 103/micro L)	1.008 (0.989–1.027)	0.379
TLC (X 103/micro L)	1.106 (0.962–1.271)	0.154
pH	0.152 (0–7207.477)	0.732
Bicarbonate (mmol/L)	1.322 (0.936–1.868)	0.113
Lactate (mmol/L)	1.929 (0.982–3.787)	0.056
PT (seconds)	0.965 (0.791–1.177)	0.728
INR	1.719 (0.149–19.812)	0.664
Blood Urea (mg/dL)	1.016 (0.992–1.04)	0.182
S. Creatinine (mg/dL)	0.949 (0.609–1.479)	0.820
AST (U/L)	0.994 (0.986–1.002)	0.171
ALT (U/L)	1.029 (0.997–1.063)	0.068
S. Albumin (g/dL)	0.246 (0.04–1.521)	0.132
Intubation requirement	20.828 (0.351–1233.096)	0.145
Oxygen requirement	45.871 (0.653–3221.18)	0.078
IV Fluid Resuscitation	0.273 (0.02–3.725)	0.330
Child Pugh Class	0.003 (0–0.289)	0.012
Need for ICU admission	0.037 (0.001–0.757)	0.032
Need for ventilator support	0.112 (0.001–8.771)	0.326
Nagelkerke R square = 0.777		

**Fig. 1:**
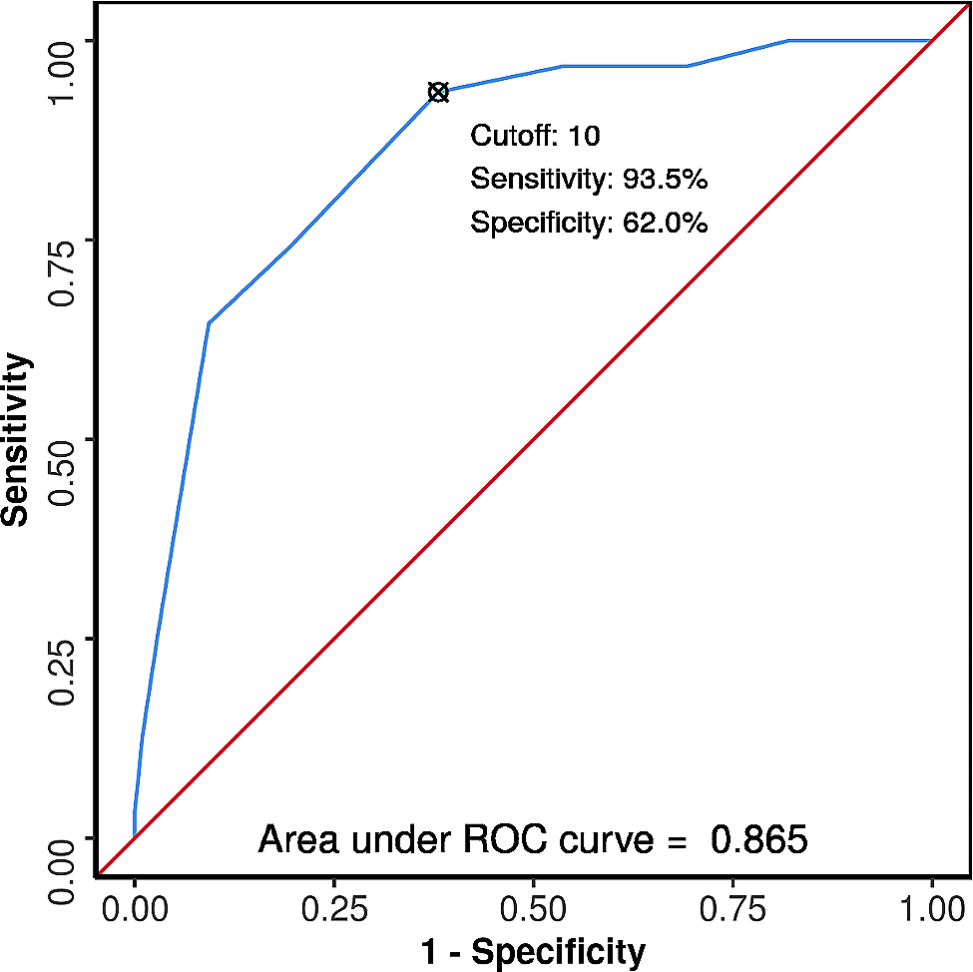
ROC curve analysis showing diagnostic performance of child-pugh score in predicting mortality during ED stay (*n* = 236)

The Child-Pugh Score has a good diagnostic performance in predicting mortality during ED stay, with an AUROC of 0.865 (95% CI: 0.801–0.928) and statistical significance (*p* < 0.001). At a cutoff of Child-Pugh Score ≥ 10, it predicts mortality during ED stay with a sensitivity of 94% and a specificity of 62%. Figure 1 illustrates the ROC curve analysis demonstrating the diagnostic performance of the Child-Pugh Score in predicting mortality during ED stay.

## Discussion

Cirrhosis and its complications cause over 40,000 fatalities in the US annually, with a mortality rate of 25.7 deaths per 100,000 persons. This makes them more deadly than kidney disorders and similar to diabetes. As cirrhosis complications continue to rise, the burden of liver disease is expected to increase significantly over time [14]. This study investigates the relation between predictive factors and mortality rates in patients with CLD seeking emergency medical attention. Similar to our study male preponderance was noted in multiple previous studies possibly due to socioeconomic factors contribute to male preponderance in ethanol consumption and access to medical care [9–14].

A study by Bhattacharyya M et al. found a comparable pattern in hemoglobin and platelet levels to our study, with 11.8% having hemoglobin levels below 6 g/dl and 28% having platelet counts below 1 lakh per milliliter of blood. Prothrombin time was abnormal in 50% of patients, and 20.7% had serum creatinine levels above 1.5 mg/dl [15]. Pathak et al. found that AST/ALT ≥ 2:1 at presentation is significantly associated with increased mortality and found a significant association between prolonged PT and increased mortality, with PT differences ≥ 5 s causing higher mortality [16].

R Maskey et al. observed that 5.7% of CLD patients attending ED left against medical advice, 67.6% were discharged, and 13.3% had fatalities during ED stay. 80% of the fatalities were caused by liver disease Child-Pugh grade C [10]. Although frequent attendance to the emergency department (more than 4 ED visits in 12 months) due to liver cirrhosis-related symptoms is common, it was not associated with increased mortality during the study period [17]. Retrospective studies in Nepal showed mortalities of 15–19%, which was comparable to our study [14, 16]. Our study included a significant proportion of rural, underprivileged, and illiterate patients who face challenges in accessibility and affordability to healthcare services due to socioeconomic inequalities. Addressing these inequalities is crucial to reducing the number of patients leaving against medical advice. Expanding government involvement in liver disease treatment could help improve access [18].

In our study, 16.9% patients died during hospital stay. Refractory septic shock was the most frequent cause of death (69.2%), followed by grade 4 hepatic encephalopathy (35.9%) and massive UGI bleeding with hypovolemic shock (23.1%).Some studies from Paris and India, the mortality rate was found to be high [13, 19]. Das et al. found that 62% of patients required mechanical ventilation and 19% needed dialysis. Of the deaths, 79% were caused by underlying illnesses such as multiple organ failure or refractory shock, while 21% were due to secondary consequences such as gastrointestinal bleeding (11%), nosocomial infection (5%), or other causes (5%) [6]. Studies conducted in Nepal revealed hepatic encephalopathy and gastrointestinal bleeding as the primary causes of mortality in the majority of cases [10, 14, 16]. In CA Onyekwere et al. found that hepatic encephalopathy had a mortality rate of 48%, with sepsis and related complications accounting for 29% of deaths [20].

Our study shows that refractory septic shock mortality rates are high, possibly due to delayed access, late presentation, and affordability of healthcare. Complications of CLD vary based on cause and geography, and infections pose a significant risk factor for death. Bacteraemia is common in cirrhotic individuals and can lead to fever, abdominal pain, dyspnoea, shock, altered consciousness, and death [17].

The Child-Pugh classification estimates the prognostic stage of cirrhotic patients using four measures: serum albumin, bilirubin, prothrombin time, and ascites. It assigns three grades: Child-Pugh grade “A” with 45% survival chance, Child-Pugh grade “B” with 20%, and Child-Pugh grade “C” with less than 20% survival [21]. The mean Child-Pugh score was 9.12 ± 2.21 in our study, and high Child-Pugh score is significantly associated with blood component transfusion, intubation, UGI endoscopy, oxygen requirement, mortality, ICU admission, ventilator support, and hospital stay.

Maskey et al. and Bhattarai et al. discovered that most cirrhotic patients had Child-Pugh Class C. Among individuals with Child-Pugh Class C, varices were found in 79%. There was significantly different in varices detection across Child-Pugh classes [10, 14]. In a study conducted in North East India by Bhattacharyya M et al., 50% of patients had Child-Pugh class C disease, indicating advanced disease [15]. Hajiani E. et al. [22] reported similar results in Iran. These findings are consistent with our research. Alam et al. reported that 30% of patients were in Child-Pugh class B, and 70% were in class C [23]. Aziz M et al. found that 39.5% of patients were in Child-Pugh class A, 35.3% were in class B, and 25.1% were in class C [24]. Khan H et al. found that the majority (83.3%) were in Child-Pugh class A, possibly because most of the cases were due viral etiology in their study [21]. Yan GZ et al. found significant differences in liver function among Child-Pugh grades; 22% of patients had cirrhosis in grade A, 41% in grade B, and 36% in grade C [25].

Presence of altered mental sensorium, increased respiratory rate, low SpO2 (%), increased heart rate, low systolic blood pressure, low diastolic blood pressure, low GCS and biochemical variables such as low random blood sugar (RBS) (mg/dL), low hemoglobin (g/dL), low hematocrit (%), decreased platelets (x 103 /micro L), increased total leukocyte count (TLC) (X 103 /micro L), low pH, low HCO3 (mmol/L), increased lactate (mmol/L), increased prothrombin time (PT) in seconds, increased INR, increased blood urea (mg/dL), increased serum creatinine (mg/dL), increased AST (U/L), increased ALT (U/L) and low serum albumin (g/dL) are found to have a statistically significant association with mortality. The parameters independently associated with mortality in our study were presence of altered mental sensorium, Glasgow coma scale, Child Pugh class and need for ICU admission.

In cirrhotic patients presenting with altered mental states, there should be a high index of suspicion of HE. A study by Rahimi et al. found that the mortality rate of individuals hospitalised with AMS was higher than those with normal mental sensorium, emphasizing the necessity of screening all patients with cirrhosis for the existence of AMS [26].

Decompensated complications in CLD raise the lactate level by increasing lactate generation and decreasing lactate clearance [27]. Lactate is a reliable predictor of both short-term and long-term mortality in patients with CLD and it is also a poor prognostic indicator for critically ill patients with Liver Cirrhosis (LC) in the Intensive Care Unit [28, 29]. In this study, the lactate level was significantly predictive of mortality in patients with CLD visiting the ED.

Hepatocytes produce albumin in the liver, which is then released into the circulation. A lower albumin concentration can impact the prognosis of CLD patients [30]. Patients with cirrhosis who present to the emergency department with creatinine levels over 1.5 mg/dL and INR levels above 1.65 have an increased risk of mortality, according to a study by R.O. Ximenes et al. [31]. In their study, Jeong et al. investigated the relationship between predictive variables and mortality in ED patients with chronic liver disease and reported that albumin, MELD score, and lactate were associated with in-hospital mortality [32]. Patients with end-stage liver disease displayed lower systolic blood pressure (SBP), elevated serum lactate, lower serum albumin, and a higher incidence of acute kidney injury (AKI), according to a study by E. Okonkwo et al. [33]. As per study by Schopis. M. et al., the blood bicarbonate level at arrival was a significant predictive predictor for poor hospital outcomes for cirrhotic patients. More extended hospital stays, and mortality were strongly associated with low serum bicarbonate levels [34]. Gessolo Lins et al.‘s study revealed that patients with renal dysfunction in progression had more fatalities among cirrhotic patients, emphasizing the necessity of a sequential assessment of renal function [35].

While most studies yielded comparable results, variances were observed in a few cases, attributable to disparities in presentation duration, etiology, follow-up procedures, and treatment compliance.

### Limitations

This study had a small sample size. A more extensive, multicentric study is required before applying the results to the general population. Studies including more variable are required to find a good fit model to predict variance in mortality. The COVID-19 pandemic during the study period might have impacted our patients’ clinical profiles. Poor follow-up compliance among patients hindered our study, and might influence the results. Also, the study did not include patients admitted to the hospital via the outpatient admission system, potentially affecting the results.

## Conclusion

Chronic liver disease and its complications are a significant health concern, with its burden likely to increase in the future. Most of the subjects were men, in their middle years, and from lower socioeconomic groups. Patients with chronic liver disease presenting to ED are typically present late, have a high inpatient mortality rate, and present with various complications. Septic shock and hepatic encephalopathy are found to be the most common causes of mortality in our study. A high Child-Pugh score is significantly associated with intubation, oxygen requirement, mortality, ICU admission and ventilator support. The parameters independently associated with mortality in our study were presence of altered mental sensorium, Glasgow coma scale, Child Pugh class and need for ICU admission. The high prevalence of critical patients visiting the emergency department, coupled with limited literature on the subject, underscores the need for additional research to identify key elements and predictive factors in CLD patients presenting to emergency departments. Understanding the presentation pattern, risk factors, and mortality predictors in patients with CLD can aid the ED physician in managing the acute event and alerting the patients about the need to receive the proper care and follow-up.

## Data Availability

The datasets used and/or analyzed during the current study are available from the corresponding author on reasonable request.
